# Taming the giant within

**DOI:** 10.1371/journal.pgen.1008098

**Published:** 2019-05-09

**Authors:** Jack R. Bateman, David J. Anderson

**Affiliations:** Biology Department, Bowdoin College, Brunswick, Maine, United States of America; Geisel School of Medicine at Dartmouth, UNITED STATES

In many eukaryotes, it is common to find genes with more DNA in their introns than in their exons. However, some genes take this to the extreme. “Intron gigantism,” a phenomenon wherein relatively small exons are interspersed with introns up to megabases long, has been observed for a handful of genes—in particular, it has been well documented for several Y chromosomal male fertility genes across diverse *Drosophila* species [1, 2]. Although this exceptional gene structure has long been recognized, it is as yet unclear whether genes with gigantic introns have any special requirements for efficient expression. Fingerhut and colleagues [3] shed light on this matter by showing that two genes, *blanks* and *heph*, are necessary for the efficient expression of *Drosophila* male fertility genes that harbor giant introns.

*Drosophila* testes can be thought of as an assembly line for sperm production, with germline stem cells at one end leading to mature sperm at the other [4]. Developing spermatocytes (SCs) undergo an extended 80–90 hour interphase, during which fertility genes harboring giant introns must be transcribed. For some of these genes, transcription creates large lampbrush-like nuclear structures, called Y-loops, that dominate much of the SC nuclear volume [1, 2, 5]. Three Y-loops have been well characterized in *Drosophila melanogaster*, with the fertility genes *kl-5*, *kl-3*, and *ks-1* being transcribed primarily in Y-loops A, B, and C, respectively.

Fingerhut and colleagues first sought to better characterize the timing of transcription for Y-loop fertility genes. They employed RNA fluorescence in situ hybridization (RNA-FISH) using probes targeting different parts of the *kl-3* and *kl-5* gene bodies—the first exons, the intronic satellite repeats, and late exons encoded hundreds of kilobases downstream from the transcription start sites. In situ signals for the first exons were seen in the nuclei of early-stage SCs, indicating that transcription initiation occurs near the beginning of SC development. Intronic transcription was observed shortly afterward, but evidence of late exon transcription was first observed in SCs that were nearly fully mature, indicating that transcription through the complete genes requires the majority of SC developmental time. Consistent with this, transcripts were not seen outside the nucleus until the final stages of SC development, when they appear as granules of spliced mRNA in the cytosol. Overall, these data suggest a need for high processivity in the RNA polymerases that carry out transcription of the repeat-rich fertility genes over the course of several days.

Intronic repeats similar to those observed in *kl-3* and *kl-5* have been previously associated with errors in transcription elongation (e.g., [6]). Fingerhut and colleagues therefore hypothesized that specific genes may be required to ensure precise transcription of Y-loop fertility genes. To test this hypothesis, they screened a set of candidate genes for those that showed potential as regulators of Y-loop transcription. Two genes, *blanks* [7] and *heph* [8], emerged as strong contenders. Specifically, green fluorescent protein (GFP)-tagged Blanks localized primarily to Y-loop B, the site of *kl-3* transcription, whereas Heph-GFP localized to Y-loops A and C, sites of *kl-5* and *ks-1* transcription, respectively. Furthermore, examination of seminal vesicles from *blanks* and *heph* mutants revealed that each mutation caused a severe drop in motile sperm count, with each mutant showing evidence of failures in axonemal development that were reminiscent of those observed in testes lacking *kl-3* or *kl-5*.

To test whether loss of *blanks* was associated with a change in Y-loop transcription, the authors assessed *kl-3* RNA in *blanks* mutant testes. RNA-FISH demonstrated that *kl-3* transcription was severely reduced relative to that observed in wild-type testes, and quantitative reverse transcription PCR indicated a drastic drop in transcriptional output beyond the first two *kl-3* exons. Consistent with these defects, cytoplasmic granules containing mature *kl-3* transcripts were rarely observed in *blanks* mutants. Conversely, transcription of *kl-5* from Y-loop A, where Blanks protein is not readily found, was only mildly affected in *blanks* mutants, and *kl-5* cytoplasmic granules were readily observed in late-stage SCs. The authors therefore concluded that *blanks* plays an important role in ensuring faithful completion of *kl-3* transcription over the course of SC development.

In contrast to *blanks’s* role in transcriptional efficacy in Y-loop B, *heph* mutants showed little change in nuclear RNA-FISH signals for *kl-5* transcripts in Y-loop A. However, late SCs in *heph* mutant testes rarely displayed cytoplasmic granules containing *kl-5*, suggesting that *heph* function may be important for a posttranscriptional step of *kl-5* RNA processing. Surprisingly, *kl-3* cytoplasmic granules were also drastically reduced in *heph* mutants, even though Heph protein localization was not observed in Y-loop B, where *kl-3* transcription takes place. This suggests a general role for Heph in processing transcripts with giant introns, perhaps reflecting that the GFP-tagged protein localization did not account for all Heph isoforms.

**Fig 1:**
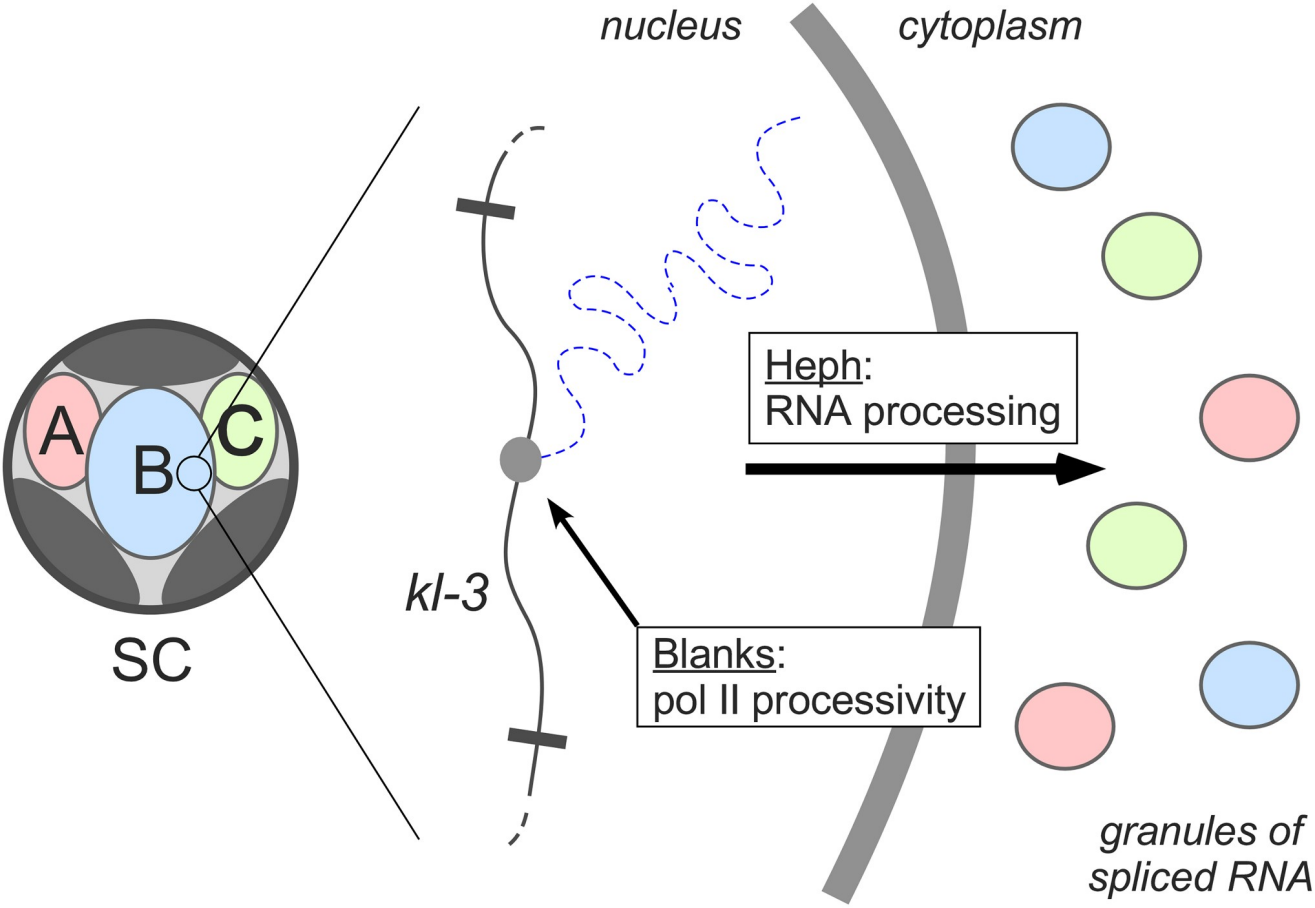
Model for a genetic pathway that accommodates intron gigantism in *Drosophila* SCs. Left, SC nucleus showing territories of paired chromosomes at the periphery (gray) and Y-loops A, B, and C. Right, detail of SC nucleus. Transcription of the repeat-rich introns of *kl-3* in Y-loop B is facilitated by Blanks. Heph is required for a posttranscriptional step, such as RNA splicing, export, or stabilization, to permit accumulation of mature transcripts from fertility genes *kl-3*, *kl-5*, and *ks-1* in cytoplasmic granules. pol II, RNA polymerase II; SC, spermatocyte.

Overall, Fingerhut and colleagues demonstrate that faithful expression of *Drosophila* fertility genes harboring giant introns requires specific gene products for both transcription and posttranscriptional processing (Fig 1). Now that this specialized transcriptional program has been uncovered by genetic analysis, mechanistic questions can begin to be addressed. Blanks has previously been characterized as an RNA-binding protein that interacts with double-stranded RNA [6], which could indicate a role for small RNA pathways in regulating *kl-3* transcription or may suggest a mechanism involving interactions between Blanks and secondary structures of repeat-rich nascent transcripts. Similarly, Heph shows homology to mammalian polypyrimidine tract-binding protein (PTB) [9, 10], which has been implicated in several steps of RNA metabolism, including splicing, stability, and export [11], each of which should be considered as a potential role for Heph. Finally, further exploration of this system may uncover other relevant players, with *blanks* and *heph* representing just part of a larger genetic program.

The study by Fingerhut and colleagues provides an early glimpse on how giant introns are dealt with during *Drosophila* spermatogenesis. However, an intriguing question remains: Why do these giant introns exist in the first place? The conservation of intron size in fertility genes across different *Drosophila* species suggests that giant introns are not simply a quirk of the *D*. *melanogaster* lineage. Given the many hours required to complete transcription of each fertility gene, the authors speculate that they could function as developmental timers, perhaps ensuring that the interphase of SC development is sufficiently long to complete the cell growth and chromosome dynamics required prior to meiosis. A similar model has been proposed to regulate expression of certain genes during the rapid cell cycles of early *Drosophila* embryonic development [12, 13]. Ultimately, the identification of new genetic players in the long-established fertility gene system will likely yield many avenues of inquiry into the impact of intron size on gene regulation.
